# The prevalence of heterotopic ossification among patients after cervical artificial disc replacement: A systematic review and meta-analysis

**DOI:** 10.1097/MD.0000000000007163

**Published:** 2017-06-16

**Authors:** Lingde Kong, Qinghua Ma, Fei Meng, Junming Cao, Kunlun Yu, Yong Shen

**Affiliations:** Department of Orthopedics, The Third Hospital of Hebei Medical University, Shijiazhuang, Hebei, P.R. China.

**Keywords:** cervical artificial disc replacement, heterotopic ossification, meta-analysis, prevalence, review

## Abstract

**Background:**

Prevalence estimates of heterotopic ossification (HO) following cervical artificial disc replacement (ADR) varied widely in previous studies. We conducted a systematic review and meta-analysis to summarize its point prevalence.

**Methods:**

Electronic searches of *PubMed*, *Web of Science*, *Embase*, and *Cochrane Library* databases were conducted to identify studies that reported prevalence of HO. Definitions of HO and severe HO were based on McAfee grading system. Random-effects model was used to estimate the pooled prevalence. We conducted subgroup analyses according to the different length of follow-up time, and performed univariate metaregression analyses to explore the effects of potential variables on the overall prevalence.

**Results:**

A total of 38 studies were included in this study. The pooled data showed that the prevalence of HO after cervical ADR within the 1 to 2 years, 2 to 5 years, and 5 to10 years of follow-up was 38.0% (95% confidence interval [CI], 30.2%–46.5%), 52.6% (95% CI, 43.1%–61.9%), and 53.6% (95% CI, 40.0%–66.7%), respectively, while the prevalence of severe HO was 10.9% (95% CI, 9.0%–13.2%), 22.2% (95% CI, 15.5%–30.7%), and 47.5% (95% CI, 30.0%–65.8%), respectively. Follow-up time was positively associated with the prevalence of severe HO (*P* < .01), and the 1-month growth of mean follow-up went with 0.63% increase of severe HO.

**Conclusion:**

This meta-analysis reported data on the prevalence of HO and severe HO after cervical ADR, and provided information on its process of development. These should be useful to enable surgeons and patients to gain a better understanding of HO after cervical ADR.

## 1 Introduction

Anterior cervical discectomy and fusion is golden standard for the treatment of cervical degenerative disc disorders with a long-term clinical success. In recent years, cervical artificial disc replacement (ADR) has become widely used in patients as a substitute for traditional fusion surgery.^[1]^ As a spinal motion-preserving technology, cervical ADR can maintain mobility and function of the index cervical segments. Clinical studies have reported good outcomes and high patient satisfaction after cervical ADR surgery.^[2]^ However, heterotopic ossification (HO) and spontaneous fusion after implantation of the cervical artificial disc have been reported, and maintenance of motion following arthroplasty can be hindered by the development of HO.^[3]^

HO is defined as formation of the bone outside the skeletal system. It is a well-known phenomenon in the field of total hip or knee joint replacement and immobilized the activity of patients after surgery.^[4,5]^ With the wide applications of artificial disc, HO has also been detected in patients after spinal ADR. In 2003, McAfee et al^[6]^ reported the occurrence of HO after lumbar ADR and proposed a classification system for HO. Since then, some spontaneous fusion following cervical ADR has also been described in the form of case reports.^[7]^ In 2005, Mehren et al^[8]^ proposed a modified classification scheme according to McAfee's study, and reported the incidence of HO after cervical ADR in a prospective multicenter study. After that, more and more studies focusing on the formation of HO after cervical ADR have been published.

Previous studies have reported the rate of HO occurrence in cervical ADR, but the outcomes were various. Some studies presented a high incidence of HO following cervical ADR,^[9,10]^ while others reported a relatively low incidence.^[11,12]^ A reliable estimate of the prevalence of postoperative HO may provide evidence for preventing, treating, and identifying causes of HO. In 2012, a meta-analysis on this topic has been performed by Chen et al,^[13]^ but their investigation only focused on the incidence of HO in the first 2 years. Besides, some relevant clinical studies have been published since their study, which may provide valuable information to us. Thus, we conducted this systematic review and meta-analysis, and tried to obtain accurate figures on the prevalence of HO within 10 years and to clarify the natural course of HO after cervical ADR.

## 2 Methods

This study is a meta-analysis, and ethics statement is not applicable. This study followed the systematic review methodology proposed in the Preferred Reporting Items for Systematic Reviews and Meta-Analyses statement.^[14]^

### 2.1 Literature search strategy

We searched *PubMed*, *Web of Science*, *Embase*, and *Cochrane Library* databases for articles published from inception to May 2016. A search strategy was developed for each database using the following search terms: (((((heterotopic ossification[MeSH Terms]) OR heterotopic ossification[Title]) OR pathologic ossification[Title])) AND (((total disc replacement[MeSH Terms]) OR disc replacement[Title]) OR disc arthroplasty[Title])) AND (((cervical vertebrae[MeSH Terms]) OR cervical[Title]) OR spinal[Title]). English language restrictions were placed on the searches or search results. The references of all publications were also retrieved to obtain possible studies.

### 2.2 Inclusion and exclusion criteria

Studies were included if they met the following inclusion criteria. First, study design was randomized controlled trials (RCTs), comparative study, cross-sectional study, or observational study. Second, sample size and point prevalence of HO were provided or could be calculated. Third, grading of HO was defined by the classification scheme proposed by McAfee et al^[6]^ Fourth, patients undergoing cervical artificial disc replacement were investigated. Fifth, patients were followed up for 1 to 10 years. Publications were excluded if they were review articles, case reports, editorials, or letters.

### 2.3 Data extraction and outcome measures

For each study included, the following information was extracted: first author, year of publication, country, type of prosthesis, study design, length of follow-up, sample size, and number of patients with HO after surgery. The most comprehensive publication was used when there were several studies involving the same population. Data were independently extracted by 2 authors, and any disagreements were resolved by discussion and consensus.

### 2.4 Diagnosis of HO

Occurrence of HO was graded on lateral radiograph, computed tomography (CT), magnetic resonance imaging (MRI), or combined methods according to the McAfee grading system^[6]^: grade 0 HO means no HO occurs on radiographs; grade 1 HO means the HO occurs as bone within soft tissue, but does not present between the planes formed by the 2 vertebral endplates and does not block motion of vertebrae; grade 2 HO means the HO occurs between the planes without blocking motion; grade 3 HO means the HO occurs between the planes and decreases range of motion (ROM) of vertebrae; grade 4 HO means the HO is severe and causes apparent bony ankylosis. As grades 3 or 4 HO can limit the ROM of the implantation segment, and may influence the clinical outcomes of cervical ADR, they were investigated independently and were defined as severe HO.

### 2.5 Assessment of methodological quality

We used the methodological scoring system described by Loney et al^[15]^ to evaluate the studies included, which is specific for studies that estimate prevalence. This scale system included 3 main items: are the study methods valid? what is the interpretation of the results? and what is the applicability of the results? This scale has a maximum score of 8 points (Table 1). The methodological quality of the studies was independently evaluated by 2 authors.

**Table 1:**
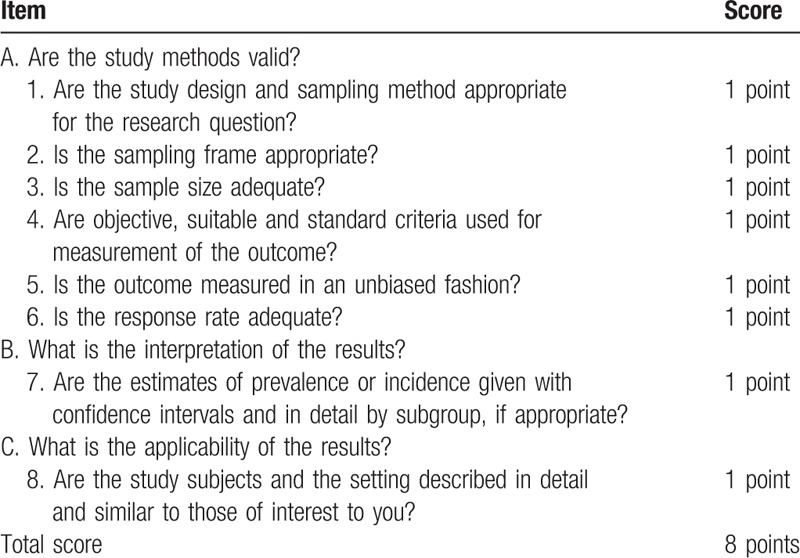
Guidelines for critically appraising prevalence studies.

### 2.6 Statistical analysis

We extracted data from each individual study, calculated the overall prevalence of HO with 95% confidence intervals (CIs), and obtained corresponding forest plots. Subgroup analysis was conducted according to the different length of follow-up: 1 to 2 years, 2 to 5 years, and 5 to 10 years. The *I*^2^ statistic and *Q* tests were used to evaluate the heterogeneity. If *I*^2^ value was >50% or *P* value was <.10, we considered that significant heterogeneity was existing. In the present meta-analysis, random-effects model was used to pool estimation of point prevalence. Furthermore, we performed univariate metaregression analyses to explore effects of the following potential variables on the overall prevalence: year of publication, Bryan Disc prosthesis, study design, mean length of follow-up, number of involved levels, and region. The influence of individual studies on the overall prevalence estimate was explored by serially excluding each study in a sensitivity analysis. Begg test, Egger test, and funnel plots were used to test the publication bias.

All analyses were performed using R version 3.2.3 (R Foundation for Statistical Computing). “Meta” package (version 4.3-2) and “metafor” package (version 1.9-8) were used. *P* <.05 was considered to be statistically significant.

## 3 Results

### 3.1 Literature search results

**Figure 1:**
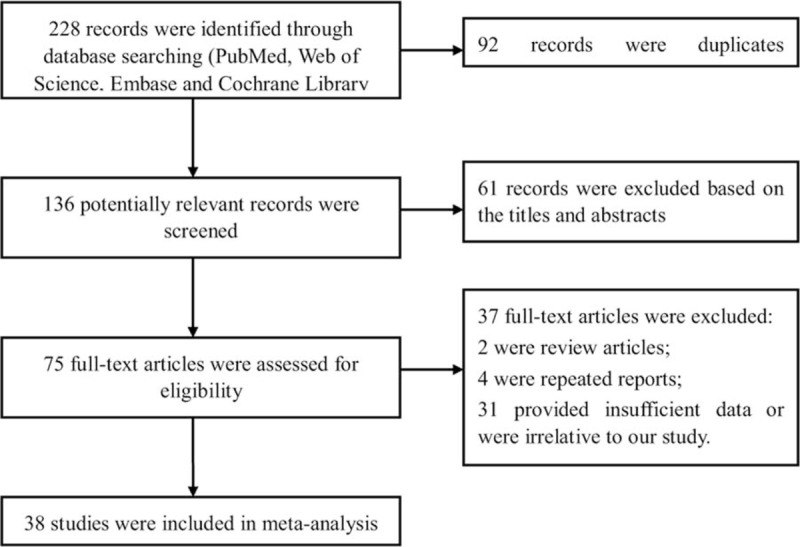
Flow diagram of study selection process in the meta-analysis.

The first searches give a total of 228 records, and 92 records were duplicates. After review of the titles and abstracts, 61 were excluded. We retrieved full articles for further assessment, and 37 records were excluded. Finally, 38 studies were included in the meta-analysis.^[3,8–12,16–47]^Figure 1 exhibits the details of screening process.

### 3.2 Study characteristics

Of the 38 studies, 33 reported HO and 30 reported severe HO. Two of them were RCTs, and the others were cohort studies. There were 2056 and 1796 patients involved, respectively. Of these studies, 25 took place in Asia, 11 in Europe, and 2 in North America. The artificial disc prosthesis included Bryan Disc, Mobi-C, ProDisc-C, and Prestige LP. Length of follow-up ranged from 1 to 10 years. The quality score of the included studies ranged from 4 to 7 points. Three studies were 7 points, 11 studies were 6 points, 17 studies were 5 points, and the left 7 studies were 4 points. Detailed information on all included studies was shown in Table 2.

**Table 2:**
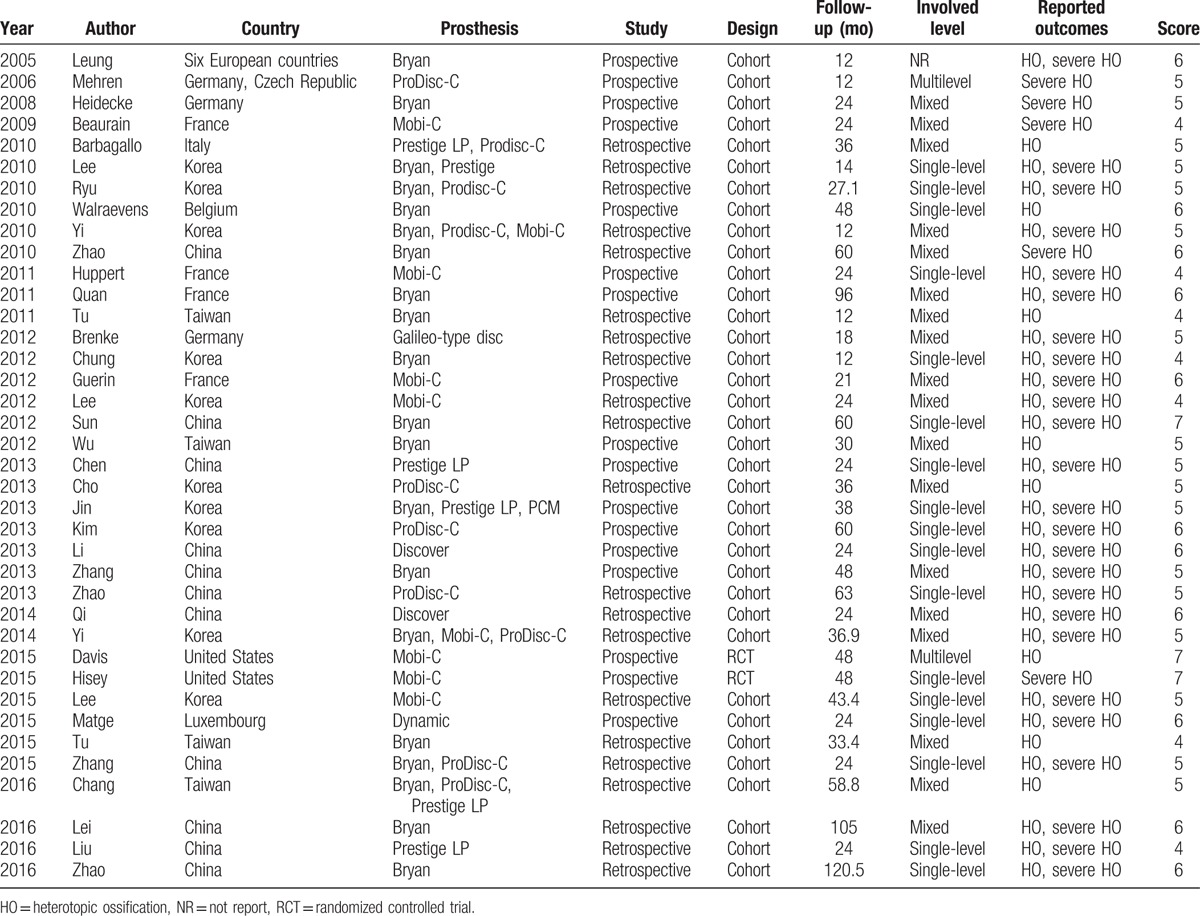
The basic characteristics of included studies.

### 3.3 Prevalence of HO

Among the studies reported the prevalence of HO after ADR, the occurrence of HO ranged from 16.1% to 85.7%, and the overall prevalence was 46.4% (95% CI, 40.1%–52.8%) by the random-effects model. There was significant heterogeneity among the studies (*I*^2^ = 85.7%; *Q* = 237.7; *P* < .01). In the cohort subgroup, the prevalence was 47.2% (95% CI, 40.9%–53.6%), and in the RCT subgroup, the prevalence was 24.8% (95% CI, 20.3%–29.8%).

**Figure 2:**
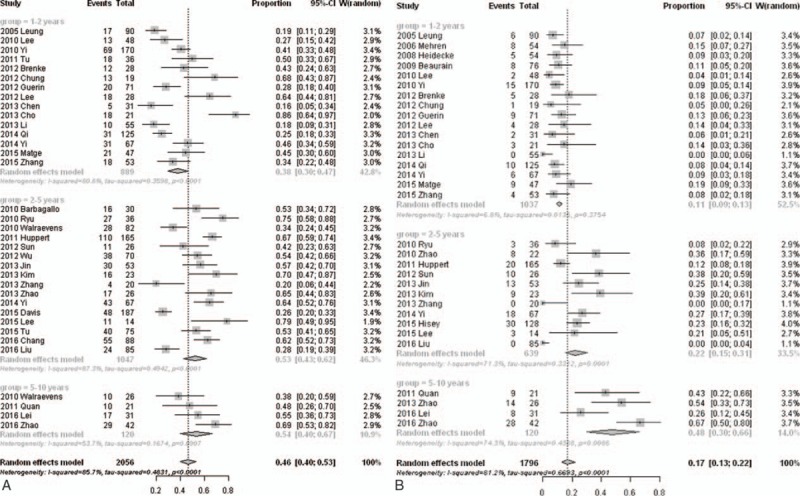
Forest plot showing subgroup analysis results of the prevalence of HO (A) and severe HO (B) after cervical artificial disc replacement.

In the 1- to 2-year subgroup, the summary prevalence of HO was 38.0% (95% CI, 30.2%–46.5%) with significant heterogeneity (*I*^2^ = 80.8%; *Q* = 73.1; *P* < .01); in the 2- to 5-year subgroup, the summary prevalence of HO was 52.6% (95% CI, 43.1%–61.9%) with significant heterogeneity (*I*^2^ = 87.3%; *Q* = 118.0; *P* < .01); and in the 5- to 10-year subgroup, the summary prevalence of HO was 53.6% (95% CI, 40.0%–66.7%) with significant heterogeneity (*I*^2^ = 53.7%; *Q* = 6.5; *P* < . 01) (Fig. 2A).

### 3.4 Prevalence of severe HO

Among the studies reported the prevalence of severe HO after ADR, the occurrence of severe HO ranged from 0% to 66.7%, and the overall prevalence was 17.0% (95% CI, 12.8%–22.2%) by the random-effects model. There was significant heterogeneity among the studies (*I*^2^ = 81.2%; *Q* = 164.8; *P* < .01).

**Figure 3:**
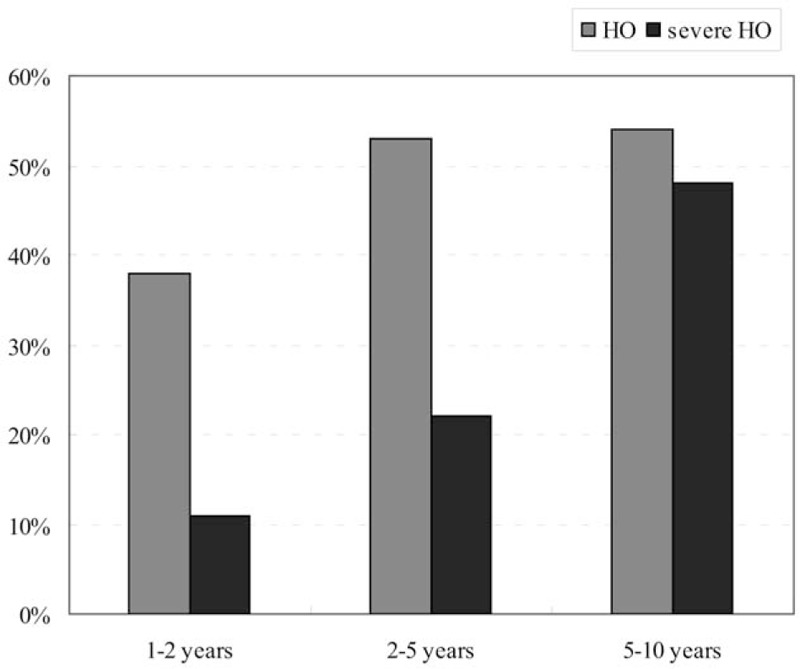
Column diagram showing the prevalence of HO and severe HO at different follow-up period.

In the 1- to 2-year subgroup, the summary prevalence of severe HO was 10.9% (95% CI, 9.0%–13.2%) without significant heterogeneity (*I*^2^ = 6.8%; *Q* = 17.2; *P* = .49); in the 2- to 5-year subgroup, the summary prevalence of severe HO was 22.2% (95% CI, 15.5%–30.7%) with significant heterogeneity (*I*^2^ = 71.3%; *Q* = 34.8; *P* < .01); and in the 5- to 10-year subgroup, the summary prevalence of severe HO was 47.5% (95% CI, 30.0%–65.8%) with significant heterogeneity (*I*^2^ = 74.3%; *Q* = 11.7; *P* < .01) (Fig. 2B). The prevalence of HO and severe HO in different follow-up time was summarized in Figure 3.

### 3.5 Metaregression analysis, publication bias, and sensitivity analysis

**Figure 4:**
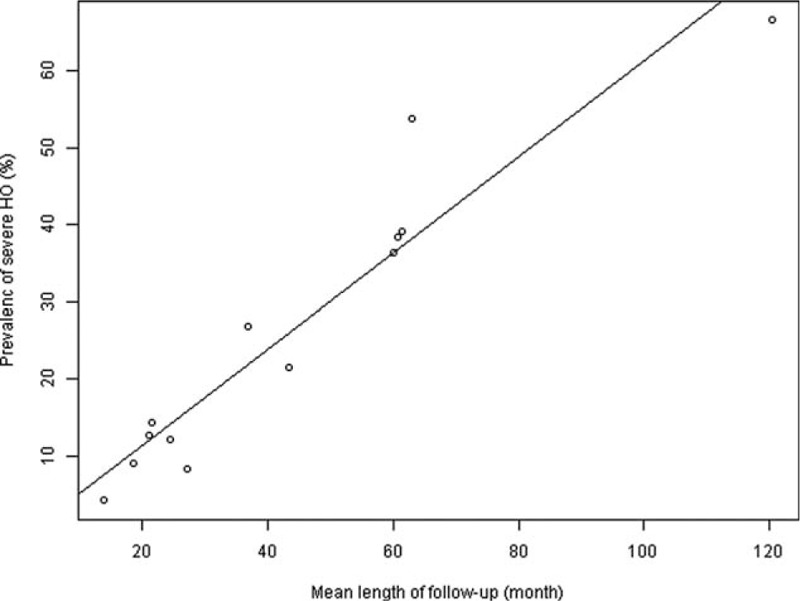
Scatter diagram showing the relationship between the prevalence of severe HO and length of follow-up time. The line represents point estimates of association between mean length of follow-up and the prevalence of severe HO; dots represent the follow-up-specific prevalence reported in different studies.

We performed univariate metaregression analyses to explore effects of the potential variables on the overall prevalence. The results of metaregression analyses are listed in Table 3. Year of publication, types of prosthesis, number of involved level, and region had no influence on the pooled prevalence. However, mean length of follow-up was positively associated with the prevalence of severe HO (*P* < .01), and the 1-month growth of mean follow-up went with 0.63% increase of severe HO (Fig. 4). Besides, the study design had an effect on the estimated prevalence of HO (*P* = .02).

**Table 3:**
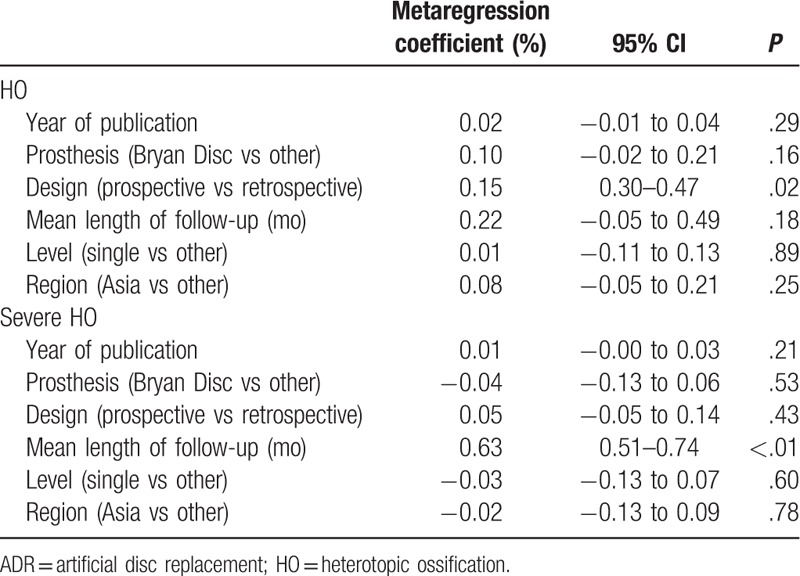
Metaregression analysis for the prevalence of HO in patients after cervical ADR.

**Figure 5:**
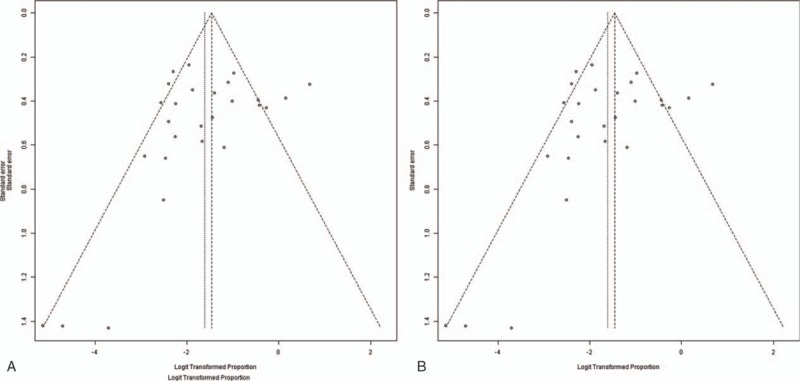
Funnel plots of the included studies in this meta-analysis for HO (A) and severe HO (B).

Sensitivity analysis, in which the meta-analyses were serially repeated after exclusion of each study, demonstrated that no individual study affected the overall prevalence estimate of HO or severe HO by >1%. Both Begg and Egger test showed negligible evidence of publication bias in the assessment of HO (Begg, *P* = .19; Egger, *P* = .29) or severe HO (Begg, *P* = .19; Egger, *P* = .12). Funnel plots were shown in Figure 5.

## 4 Discussion

Occurrence of HO is an inevitable postoperative complication after cervical ADR, and can decrease the ROM of index segment, which is contrary to the fundamental goal of artificial disc. Previous studies reported various results on the occurrence of HO. Leung et al^[16]^ presented 17.8% of HO occurrence in studied patients at 12 months of follow-up, but Lee et al^[41]^ reported 78.6% patients exhibited HO at a mean follow-up period of 43.4 months. In the study conducted by Yang et al,^[48]^ the incidence of HO was up to 90%, but their results were based on a 30-year follow-up. There is a hypothesis that HO is not a static, but rather a dynamic and progressive phenomenon that is affected by environment.^[38]^ If so, different length of follow-up would definitely affect the final results. In our study, the results of HO and severe HO were grouped into different subgroups, and the pooled data showed that the prevalence of HO after cervical ADR within the 1 to 2 years, 2 to 5 years, and 5 to 10 years of follow-up was 38.0%, 52.6%, and 53.6%, respectively, while the prevalence of severe HO was 10.9%, 22.2%, and 47.5%, respectively.

Another reason explaining the different rates of HO in different studies would be interobserver error. In the process of investigation of images, detection sensitivity and determination of HO would be different among the authors in the various institutes. By this reason, it would be important to make consensus on the definition of HO. In our meta-analysis, all included studies defined HO on the basis of the McAfee classification system.^[6]^ A standard method can effectively decrease the heterogeneity among the studies.

The prevalence of both HO and severe HO showed a trend of progression. After univariate metaregression analyses, we found a linear relationship between mean length of follow-up and the prevalence of severe HO. Within 10 years, the 1-month growth of mean follow-up went with 0.63% increase of severe HO. However, this linear relationship was not shown in the investigation of HO prevalence. From Figure 3, we found that the prevalence of HO increased gradually in short (1–2 years) and mid-term (2–5 years) follow-up, but this prevalence did not increase significantly in the long-term (5–10 years) follow-up. Although new HO did not develop any more, the progression of HO did not stop, and HO still progressed gradually into severe HO in the long-term follow-up, which can limit the movement of index level and may influence the clinical outcomes of cervical ADR.

The factors associated with HO occurrence have not been clarified. Yi et al^[22]^ found the difference in HO occurrence according to different types of prosthesis. The Bryan Disc, which has the most unconstrained motion, showed significantly lower incidence of HO occurrence in comparison with other prosthesis. They proposed that differences in the design, biomechanical property, and prosthesis-specific endplate articulation component could contribute to the formation of HO. However, in our study, the Bryan Dis prosthesis did not seem to be associated with low prevalence of HO or severe HO. As great heterogeneity was existed in this study, effect of prosthesis on the prevalence of HO may not display. The influence of prosthesis on the occurrence of HO needs to be elucidated in the future study.

Several limitations should be considered when interpreting the findings of this study. First, there were studies that showed a significant relationship between HO in cervical ADR patients and male sex, old age, multisegmental operation, and so on,^[16]^ but our meta-analysis cannot collect enough information and number of studies regarding these potential moderating factors. Second, not all of the included studies were designed for the prevalence study. Some of them did not provide detailed characteristics of patients with HO, and this may led to the impreciseness of the pooled data. Third, the HO determination was uniformed by McAfee classification, but the methods of detecting of HO varied. Some studies used radiography, while some studies used CT or MRI. The interobserver error was inevitable. Finally, there were still considerable differences across studies, like study design, prosthesis, and involved levels. These differences can increase heterogeneity and have an effect on the final results. Although potential sources of heterogeneity were explored, we still cannot explain the heterogeneity sufficiently.

In conclusion, this meta-analysis provides detailed information on the prevalence of HO and severe HO after cervical ADR. In the long-term follow-up, though new HO did not develop too much, the progression of HO did not stop. This information should be useful to enable surgeons and patients to gain a better understanding of HO during follow-up. However, because of the heterogeneity among the studies, the results of this meta-analysis should be interpreted with caution.

## Acknowledgments

We are very grateful for many helpful comments by reviewers and editors on an earlier version of this manuscript.
